# Supplementary material for: A scalable screening of E. coli strains for recombinant protein expression
Source: PLoS One. 2022 Jul 25;17(7):e0271403. doi: 10.1371/journal.pone.0271403 (PMC9312941; doi:10.1371/journal.pone.0271403)
Supplement: S1 Fig — For most of the targets (e.g., T11, T4, T2, T6, T14), only marginal soluble expression could be detected by the gel intensities. For other targets, such as T3, T5, T8, for example, some soluble protein was recovered in this strain. (DOCX) [file pone.0271403.s001.docx]

A Scalable Screening of *E. coli* Strains for Recombinant Protein Expression

Luana G. Morão^*a¶^, Lívia R. Manzine^a¶^, Lívia Oliveira D. Clementino^a^, Carsten Wrenger^b^ and Alessandro S. Nascimento^a^*.

a. Pólo TerRa, São Carlos Institute of Physics, University of São Paulo. Av. João Dagnone, 1100, Jd. Santa Angelina, 13563-120. São Carlos, SP. Brazil.

b. Unit for Drug Discovery, Department of Parasitology, Institute of Biomedical Sciences, University of São Paulo, Av. Prof. Lineu Prestes 1374, 05508-000 São Paulo-SP, Brazil.

^¶^ These authors contributed equally to this work.

* Corresponding authors: Phone: +55-16-3364-8075. Email: [asnascimento@ifsc.usp.br](mailto:asnascimento@ifsc.usp.br) (ASN) or [luanagm_bio@yahoo.com.br](mailto:luanagm_bio@yahoo.com.br) (LGM).


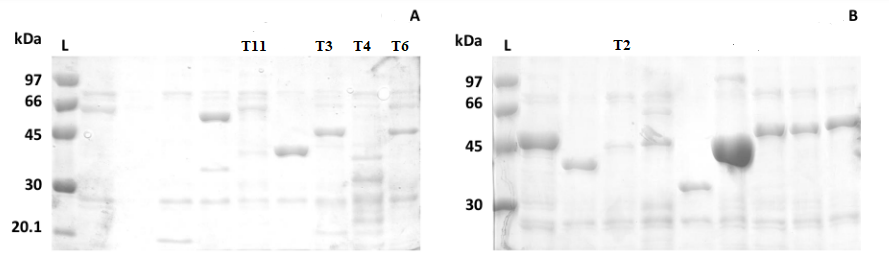


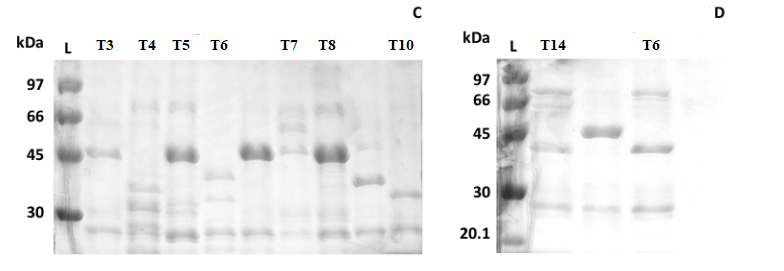


**Supplementary Figure 1**. SDS gels with the soluble protein recovered after expression and affinity purification of selected targets in *E. coli* Rosetta 2 strain. For most of the targets (*e.g*., **T11**, **T4**, **T2**, **T6**, **T14**), only marginal soluble expression could be detected by the gel intensities. For other targets, such as **T3**, **T5**, **T8**, for example, some soluble protein was recovered in this strain.
