# Supplementary figures and images for: A scalable screening of E. coli strains for recombinant protein expression
Source: PLoS One. 2022 Jul 25;17(7):e0271403. doi: 10.1371/journal.pone.0271403 (PMC9312941; doi:10.1371/journal.pone.0271403)

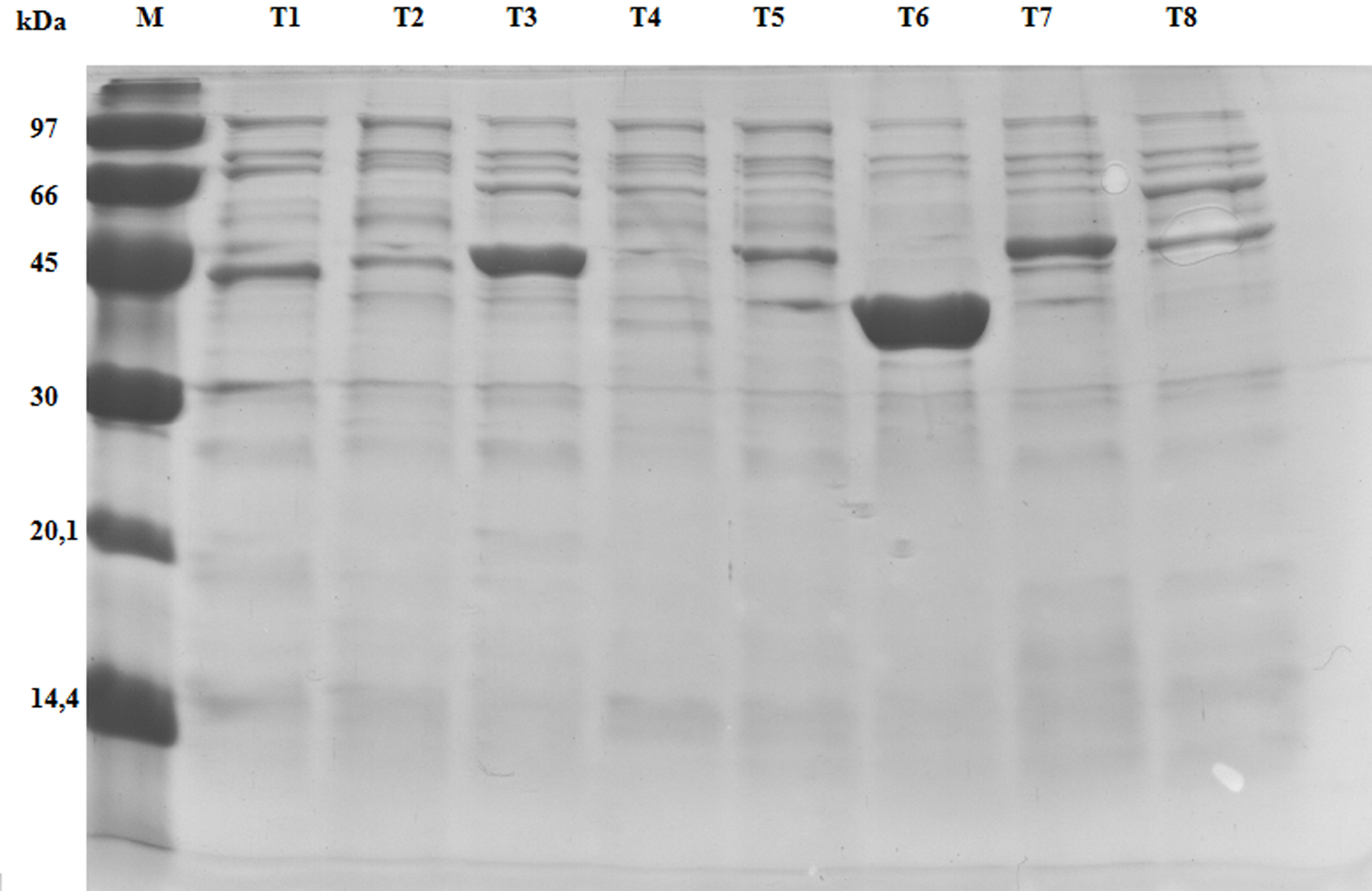

Supplement: S1 Raw images — (ZIP) [file pone.0271403.s002.zip › SI_raw_image_ARCTIC_GEL_1.tif]

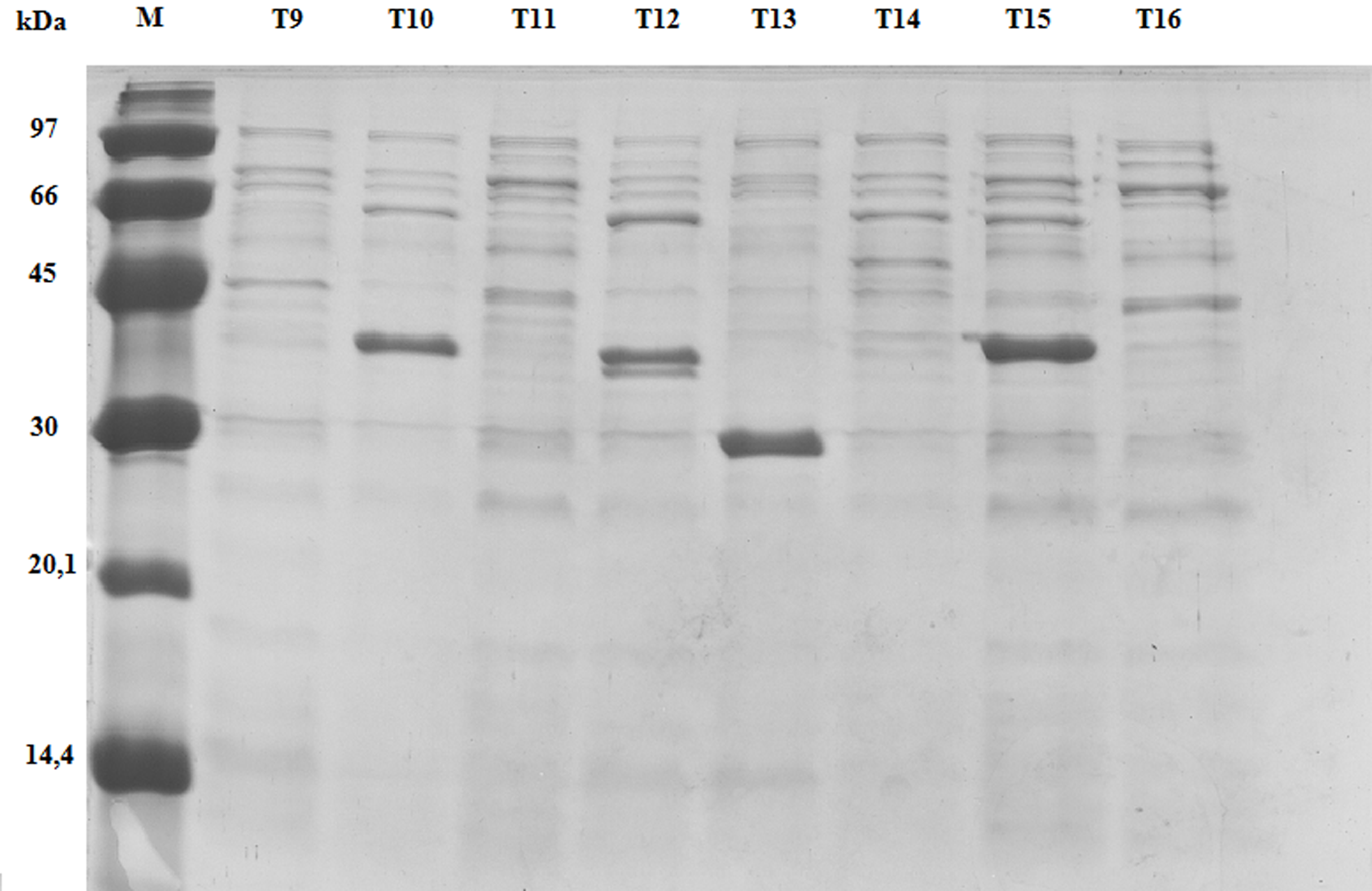

Supplement: S1 Raw images — (ZIP) [file pone.0271403.s002.zip › SI_raw_image_ARCTIC_GEL_2.tif]

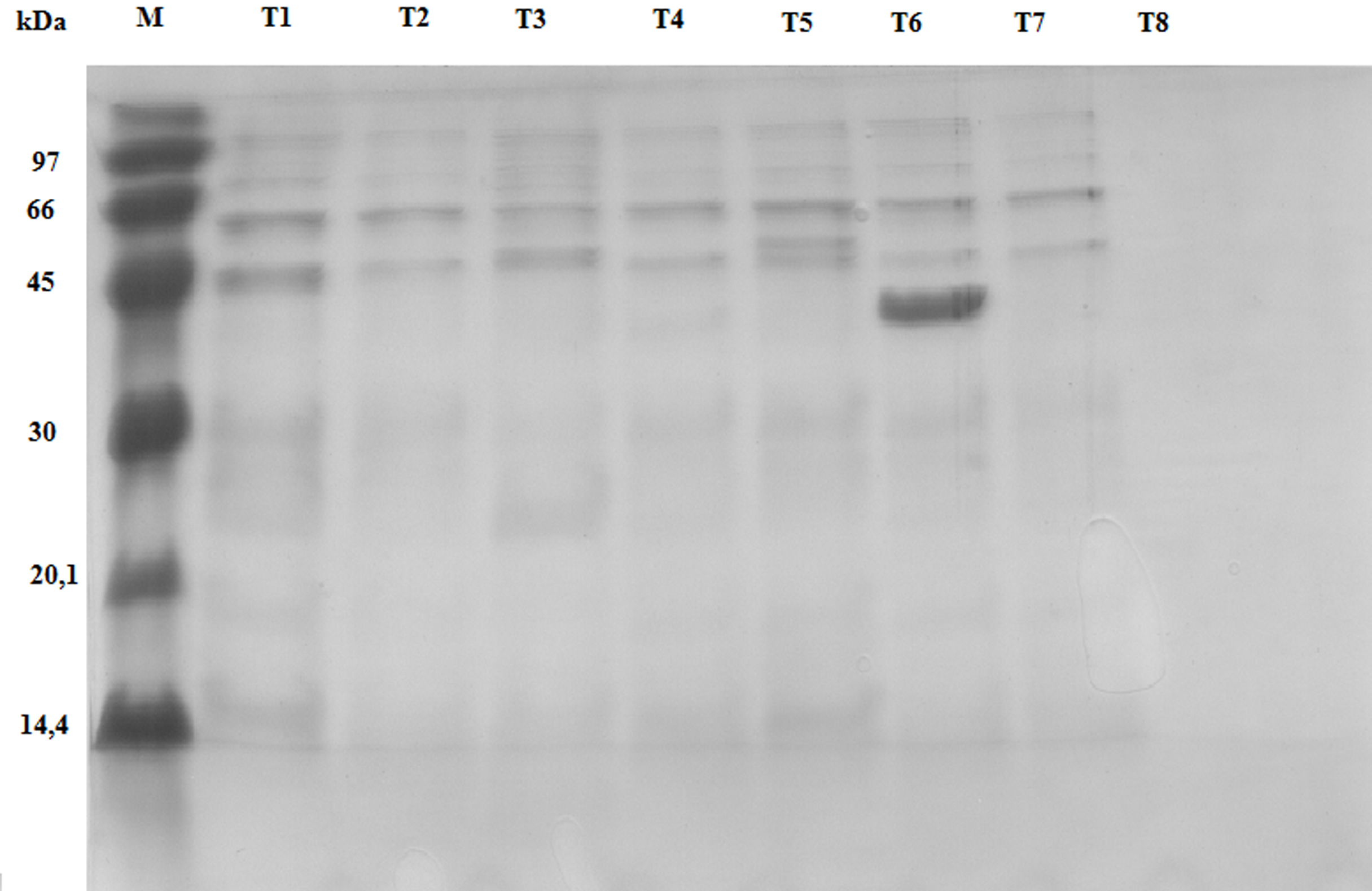

Supplement: S1 Raw images — (ZIP) [file pone.0271403.s002.zip › SI_raw_image_GAMI2_GEL_1.tif]

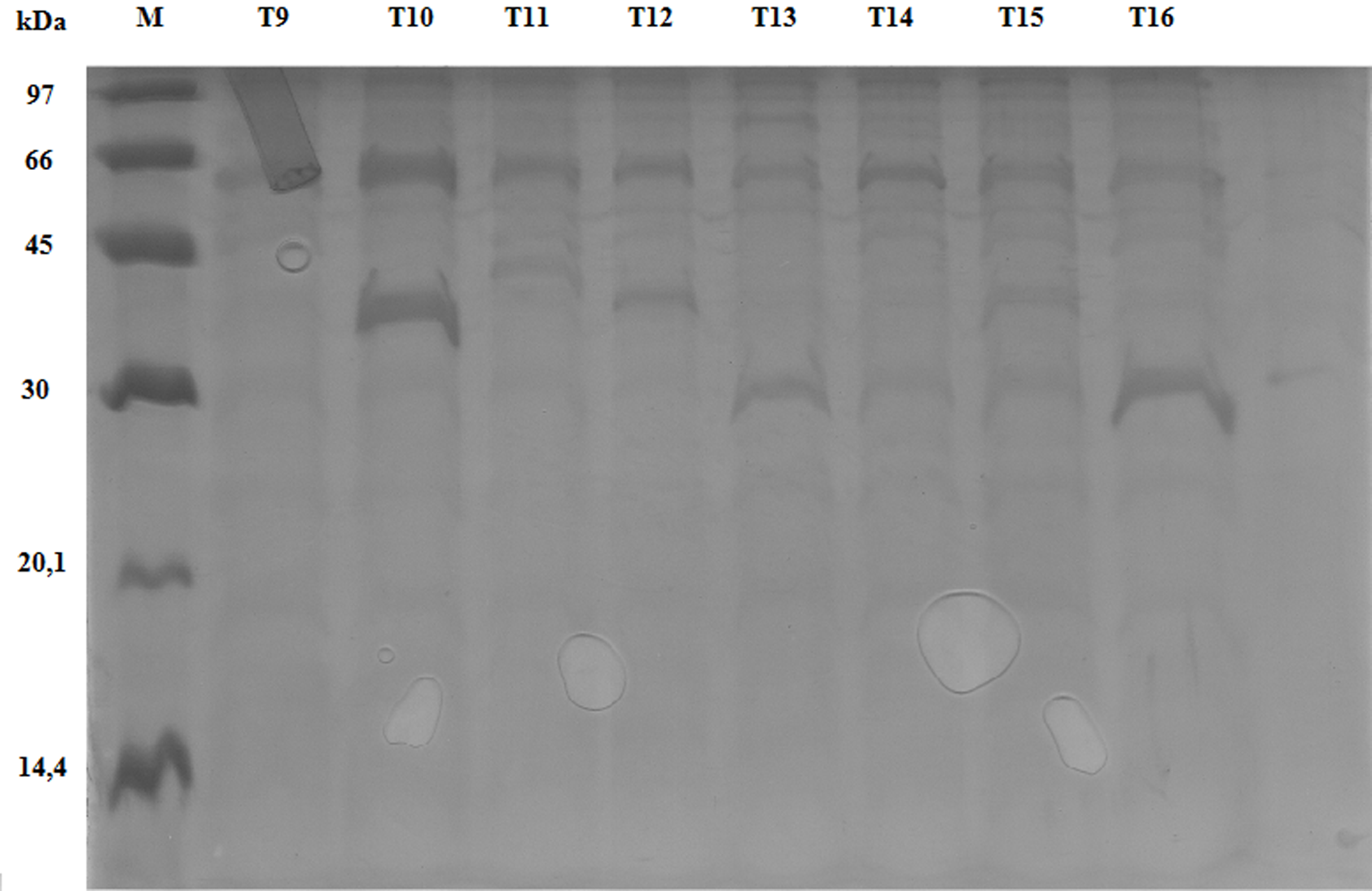

Supplement: S1 Raw images — (ZIP) [file pone.0271403.s002.zip › SI_raw_image_GAMI2_GEL_2.tif]

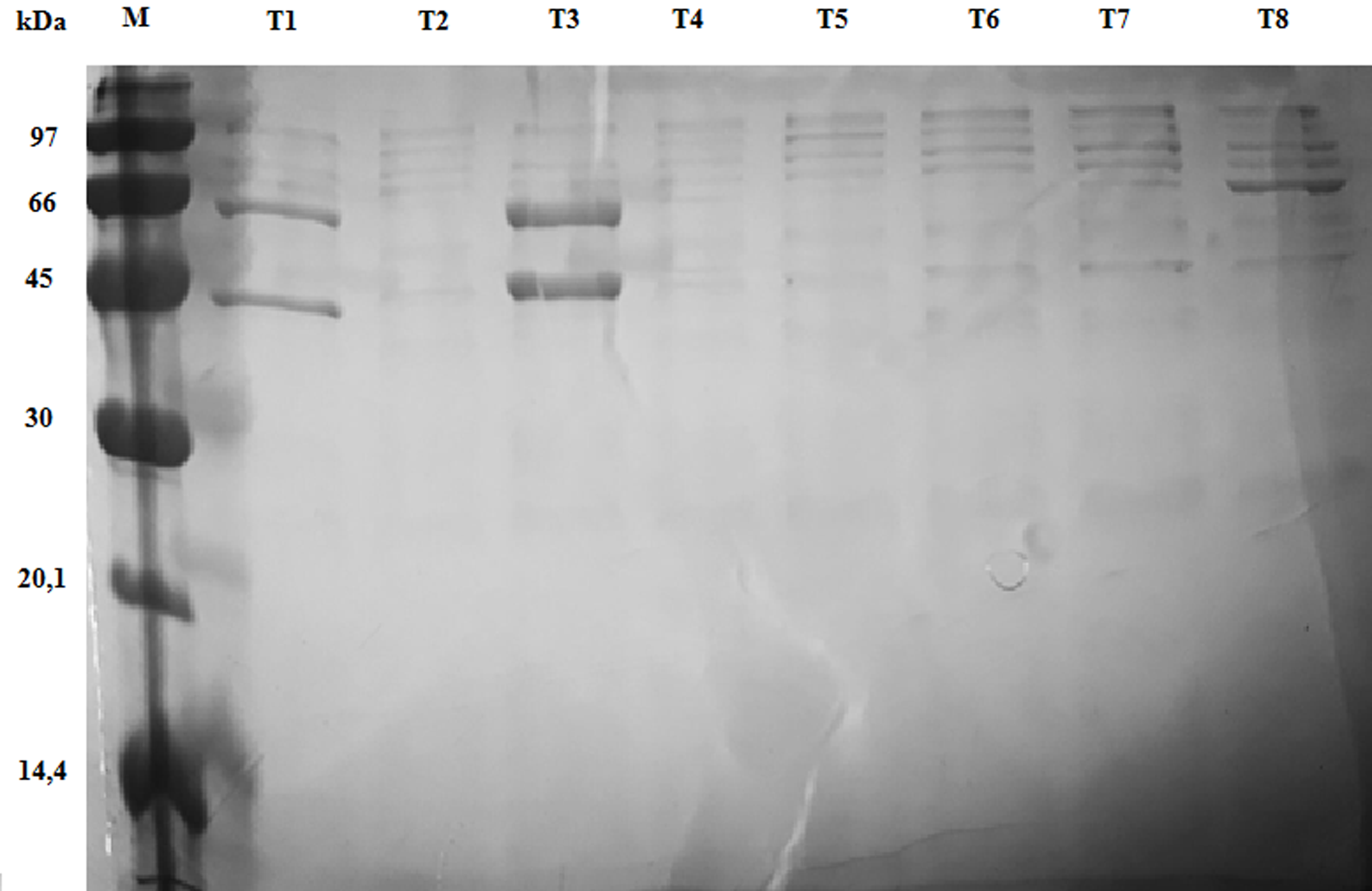

Supplement: S1 Raw images — (ZIP) [file pone.0271403.s002.zip › SI_raw_image_GROE_GEL_1.tif]

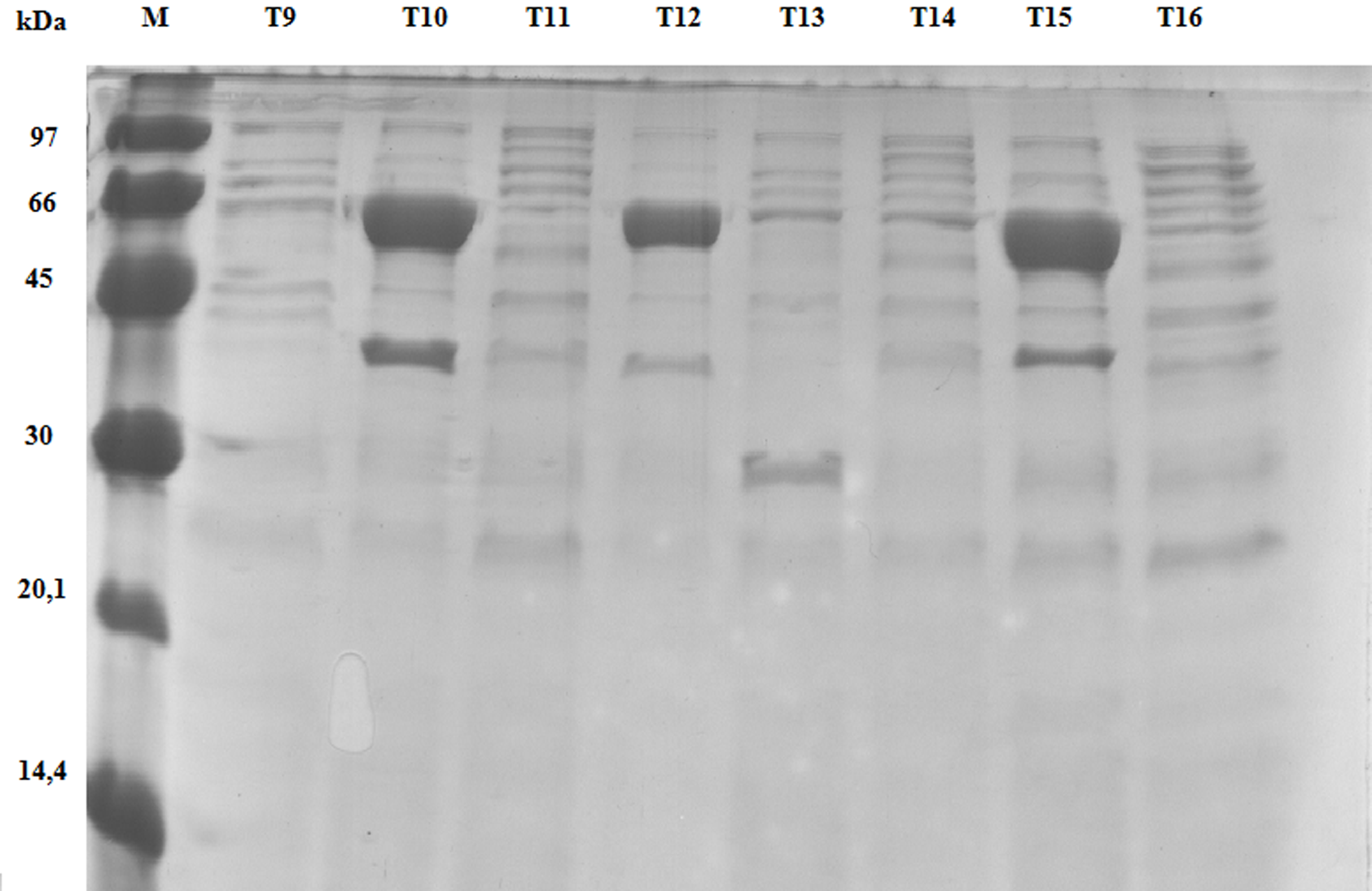

Supplement: S1 Raw images — (ZIP) [file pone.0271403.s002.zip › SI_raw_image_GROE_GEL_2.tif]

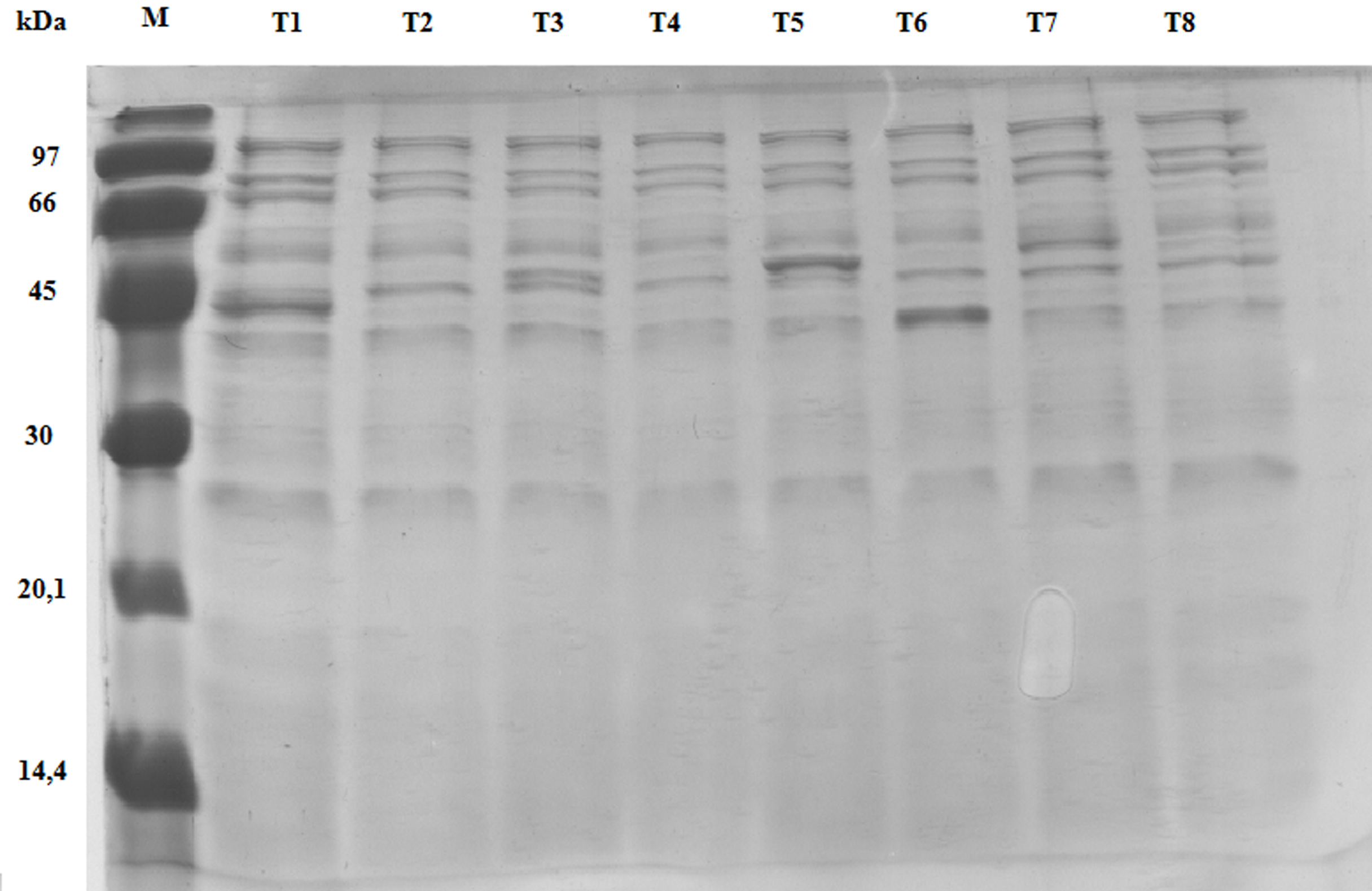

Supplement: S1 Raw images — (ZIP) [file pone.0271403.s002.zip › SI_raw_image_LEMO21_GEL_1.tif]

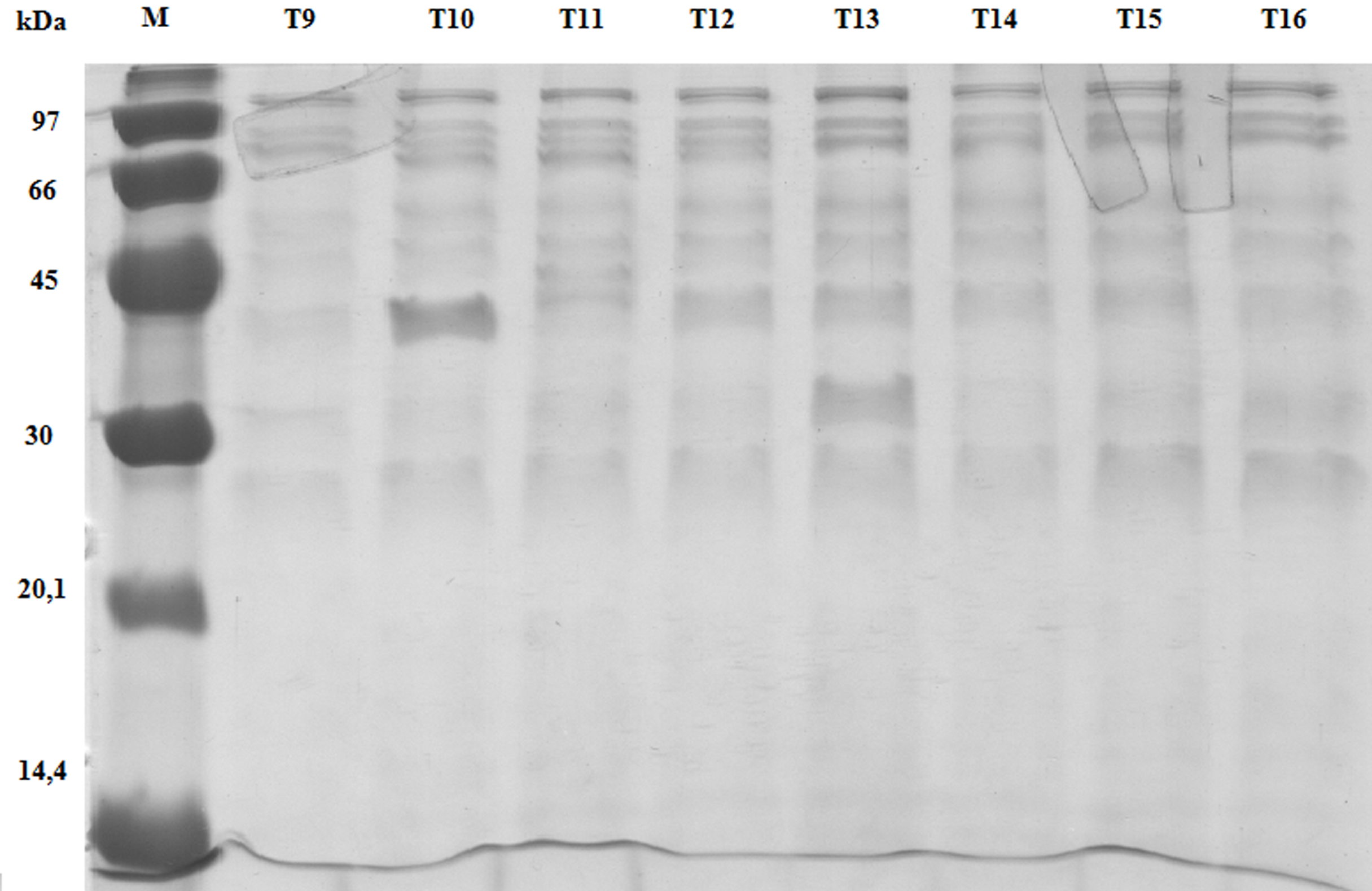

Supplement: S1 Raw images — (ZIP) [file pone.0271403.s002.zip › SI_raw_image_LEMO21_GEL_2.tif]
